# Clinical Outcomes after Liver Transplantation for Hepatorenal Syndrome: A Systematic Review and Meta-Analysis

**DOI:** 10.1155/2018/5362810

**Published:** 2018-05-24

**Authors:** Piyapon Utako, Thapanakul Emyoo, Thunyarat Anothaisintawee, Noriyo Yamashiki, Ammarin Thakkinstian, Abhasnee Sobhonslidsuk

**Affiliations:** ^1^Division of Gastroenterology and Hepatology, Department of Medicine, Faculty of Medicine Ramathibodi Hospital, Mahidol University, Bangkok, Thailand; ^2^Department of Family Medicine, Faculty of Medicine Ramathibodi Hospital, Mahidol University, Bangkok, Thailand; ^3^Section for Clinical Epidemiology and Biostatistics, Faculty of Medicine Ramathibodi Hospital, Mahidol University, Bangkok, Thailand; ^4^Organ Transplantation Unit, Kyoto University Hospital, Kyoto, Japan

## Abstract

**Aims:**

Hepatorenal syndrome (HRS) decreases survival of cirrhotic patients. The outcomes of HRS after liver transplantation (LT) were inconsistently reported. We conducted a systematic review and meta-analysis study to estimate the post-LT rates of death and HRS reversal.

**Methods:**

A thorough search of literatures was performed on PubMed, Scopus, and conference abstracts for reports on post-LT survival and HRS reversal. Data for the posttransplant rates of HRS reversal, death, and acute rejection were extracted. The rates were pooled using inverse variance method if there was no heterogeneity between studies. Otherwise, the random effect model was applied.

**Results:**

Twenty studies were included. Pooling HRS reversal rates indicated high heterogeneity with a pooled rate of 0.834 (95% CI: 0.709–0.933). The pooled overall death rates for HRS and non-HRS after LT were 0.25 (95% confidence interval (CI): 0.18–0.33) and 0.19 (95% CI: 0.14–0.26). The risk ratio of death between HRS and non-HRS patients was 1.29 (95% CI: 1.14–1.47, *P* < 0.001). The probability of death at 1, 3, and 5 years tended to be higher among HRS.

**Conclusions:**

HRS is reversible in about 83% of patients after LT. However, the posttransplant mortality rate of HRS patients is still increased.

## 1. Introduction

The annual mortality rates of patients with cirrhosis vary from as low as 5.4% in cases of compensated cirrhosis to 20.2% in decompensated cases [1]. Hepatorenal syndrome (HRS) is a functional renal failure that occurs in patients with decompensated cirrhosis after precipitating acute events such as bacterial infection. The primary features of HRS include impaired kidney functions, intense changes in the sympathetic nervous system and renin-angiotensin system, and extreme alterations in cardiovascular function. Renal dysfunction associated with HRS causes a lower survival in patients with decompensated cirrhosis. In 1996, the International Ascites Club (IAC) proposed diagnostic criteria of HRS that were adopted worldwide [2]. These criteria were subsequently revised in 2007 and 2015 [3, 4]. The IAC classifies HRS into two types according to the severity and the rate of disease progression [5]. Type I HRS manifests as acute renal failure and is characterized by a more aggressive clinical course, while type II HRS involves slow, progressive chronic renal failure associated with massive ascites.

The initial management of HRS generally includes supportive care and concurrent infusion with a vasoconstrictor and albumin. However, the pharmacological approach is not a definite treatment of HRS, and it has transient effects on HRS reversal in some patients. The rates of recurrent HRS after completion of pharmacotherapy ranged from 20% to 55% [3, 6]. Liver transplantation (LT) has been the optimal treatment for HRS [5]. Some studies have reported long-term outcomes of HRS after LT that include HRS reversal and improved survival among these patients [7]. However, the reported rates of HRS reversal and posttransplant survival have been inconsistent across studies, countries, and patient characteristics. Therefore, we conducted a systematic review and meta-analysis to estimate the outcomes of HRS reversal, death, and acute cellular rejection (ACR) rate in HRS patients who underwent LT.

## 2. Materials and Methods

This meta-analysis was conducted according to the Preferred Reporting Items for Systematic Reviews and Meta-Analysis (PRISMA) guidelines [8]. The review protocol was registered at PROSPERO (the International Prospective Register of Systematic Reviews [9]; registration number**:** CRD42016033164).

### 2.1. Search Strategy

Two investigators (P.U. and T.E.) independently identified relevant publications on the MEDLINE and Scopus databases using the PubMed and Scopus search engines. The search was restricted to manuscripts that had been published between January 1, 1996, and June 30, 2017. The references of the selected articles were also reviewed. In addition, abstracts from the European Association for the Study of the Liver, the American Association for the Study of Liver Disease, and Digestive Disease Week meetings were also examined. The following search terms were constructed based on the type of patients, intervention/exposure, and outcome: (Patients: “HRS” or “hepatorenal syndrome” or “renal failure” or “kidney failure” or “kidney injury”) AND (Patients: “Cirrhosis” or “liver failure” or “hepatic failure” or “hepatic decompensation” or “end-stage liver disease”) AND (Exposure: “liver transplantation”) AND (Outcomes: “survival” or “reversal” or “reversibility” or “mortality” or “death” or “graft loss” or “graft rejection” or “graft failure” or “post transplantation”).

### 2.2. Study Selection

The investigators (P.U. and T.E.) independently assessed the potential relevant studies. The studies were screened for relevance based on their titles and abstracts. The full articles were retrieved if a decision could not be made based on the abstracts. Full papers were examined and read thoroughly. A third investigator (A.S.) provided consensus and judgment in the case of disagreements in paper selection.

*Inclusion Criteria*. Studies published in any language were eligible if they satisfied all of the following criteria:

- The study design involved a prospective/retrospective cohort or a randomized, controlled trial (RCT) of HRS patients that reported an outcome of interest after LT with or without comparing them with non-HRS patients.
- The study patients were adults aged 18 years or older who were diagnosed with cirrhosis with HRS and underwent LT.
- The study reported any of the following clinical outcomes: survival/death, HRS reversal, or acute rejection rate after LT.

*Exclusion Criteria. *The exclusion criteria were as follows:

- Combined liver and kidney transplantation (CLKT) existed.
- Language translation was not possible.
- Insufficient data were obtained after attempting to contact the corresponding author three times over a period of 2 months.

### 2.3. Definition of HRS

HRS was defined according to the included studies, which mostly used the IAC criteria of HRS proposed in 1996 [2] and/or 2007 [3]. Two types of HRS were defined, i.e., type I and type II HRS. Type I HRS was defined as acute kidney injury that occurred in cirrhotic patients with ascites [10]. The acute kidney injury was known or at least presumed to have the following criteria: an absence of shock and hypovolemia, no current or recent nephrotoxic drug treatment, and an absence of parenchymal renal disease [2, 3]. Type II HRS was defined as having slow progressive decline of renal function, which often exists with refractory ascites [2, 3].

### 2.4. Outcomes of Interest

The outcomes of interest included HRS reversal, death, and graft rejection. These outcomes were defined according to each individual study.

### 2.5. Data Extraction

Data obtained from each study were independently extracted by two of the investigators (P.U. and T.E.) using standardized extraction forms. The characteristics of the studies and patients included the setting and study design, number of study patients, mean age, sex, mean score on the Model of End-stage Liver Disease (MELD), cause of cirrhosis, and laboratory data. In addition, data used for pooling outcomes of interests were extracted, including death/survival, HRS reversal, graft failure, and graft rejection. The corresponding authors of the studies were contacted if there was any missing information.

### 2.6. Quality and Risk-of-Bias Assessment

All of the selected studies were independently assessed for risk of bias by the investigators (P.U. and T.E.). Quality was assessed using the Newcastle-Ottawa scale for cohort studies [11]. The quality criteria included representativeness of the exposed cohort, selection of the nonexposed cohort, ascertainment of exposure, demonstration that the outcome of interest was not present at the start of the study, comparability of cohorts based on the design or analysis, and assessment of the outcomes. Disagreement was resolved by discussion and consensus with a third investigator (A.S.).

### 2.7. Statistical Analysis

Frequency data were extracted from individual studies for the outcomes of interest (HRS reversal, death, and graft failure) at the end of each study or at each distinct time period (e.g., 1 year, 5 years). Most of the included studies reported data for the comparison of death rates between the HRS and non-HRS groups. Therefore, these data were expanded from aggregated data into individual patient data. A mixed-effect Poisson regression was then applied to estimate and compare death rates between groups. A relative risk ratio was estimated along with the 95% confidence interval (95% CI).

For the studies with groups of HRS patients, we estimated the rates of reversal and graft failure along with their variances. The rates were then pooled across the studies using an inverse variance method [12]. The random effect model was applied instead, if heterogeneity between studies was presented. Heterogeneity was assessed using a *Q* test, and the degree of heterogeneity was quantified using *I*^2^. Heterogeneity was considered present if the* P* value of the *Q* test was less than 0.10 or if *I*^2^ exceeded 25%. The sources of heterogeneity were then explored using a metaregression if the data of the covariables were available. A subgroup analysis was then performed accordingly.

Publication bias was assessed using Egger's test and a funnel plot. The analyses were performed using STATA software version 14 (StataCorp, Texas). A two-sided test with *P* < 0.05 was considered to be statistically significant except for the heterogeneity test, for which a one-sided test with *P* < 0.10 was used.

## 3. Results

### 3.1. Study Identification and Characteristics

**Figure 1:**
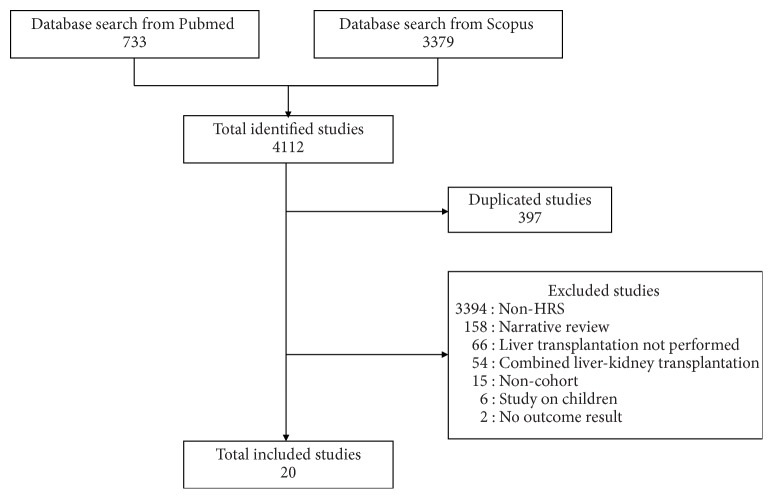
Flowchart detailing study isolation and selection.

A total of 4,112 studies were identified from the PubMed and Scopus databases, and 397 duplicate studies were removed. After screening the titles or abstracts and reading the full papers, 3,715 studies were excluded for being non-HRS studies (3,394), noncohort studies (15), narrative reviews (158), studies that lacked LT (66), studies performed on children (6), studies that included CLKT (54), and studies that presented no outcome of interest (2). Ultimately, 20 studies were included [7, 13–31] (Figure 1).

The characteristics of the studies are presented in Table 1. Thirteen, five, and two studies used the IAC criteria of HRS proposed in 1996, 2007, and 1996 together with 2007, respectively. All of the studies were cohorts except for one, which was an RCT [14]. Eleven studies were double-arm conducted. The sample sizes of the studies ranged from 8 to 130 with a total of 942 patients with HRS included. Among the 19 cohorts, 9 were prospective data collections and 10 were retrospective data collections. Most of the studies (70%) were conducted in Western countries, and 6 studies were conducted in Asia (mainly in Korea and China). The types of LT included deceased donor liver transplantation (DDLT) in 15 studies, living donor liver transplantation (LDLT) in 4 studies, and both DDLT and LDLT in 1 study. The mean age of the patient cohorts in the studies varied from 46 to 58 years, the mean MELD score varied from 21 to 43, and mean serum creatinine level prior to LT varied from 1.8 to 3.3 mg/dL. Among the 20 studies, 17 studies reported death rates, 8 reported reversal rates, and 3 reported ACR rates. These outcomes were pooled and described.

### 3.2. Risk of Bias

Two authors (P.U. and T.E.) independently assessed the risk of bias of the included studies. A few disagreements occurred between the two reviewers, and they were resolved by discussion. Most of the included studies were considered to have moderate risk of bias based on the Newcastle-Ottawa scale (Table S1).

### 3.3. Incidence of HRS Reversal

**Figure 2:**
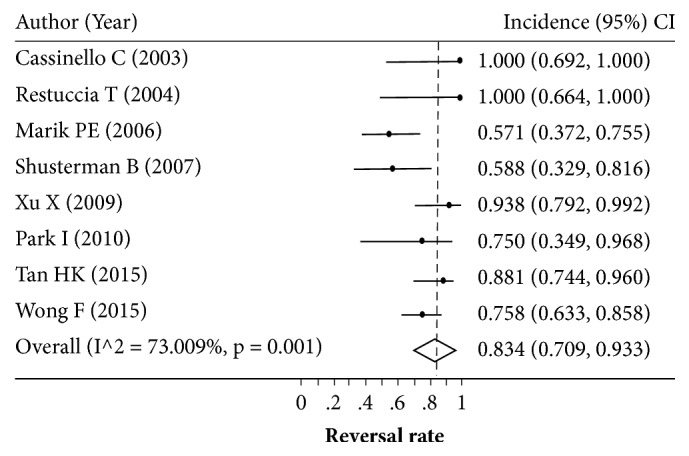
Pooling incidence of hepatorenal syndrome reversal.

**Figure 3:**
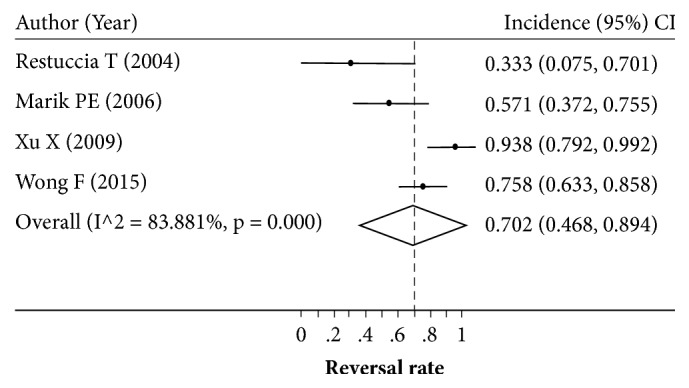
Pooling incidence of type I hepatorenal syndrome reversal.

**Figure 4:**
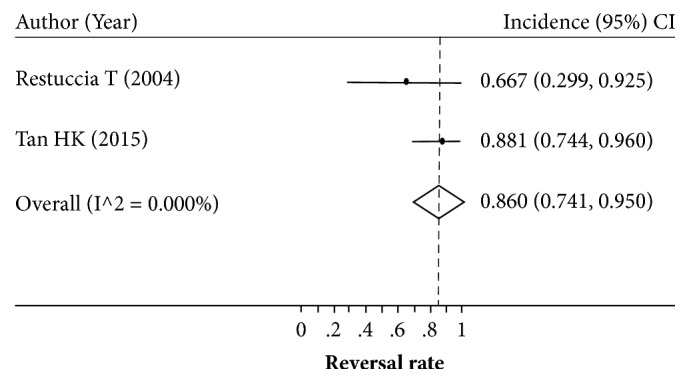
Pooling incidence of type II hepatorenal syndrome reversal.

Eight studies with sample sizes of 8–42 patients reported the reversal rate after LT [7, 15, 19, 21, 23, 28, 29, 31]. The HRS reversal rate varied across studies (0.571–1.000) with a degree of heterogeneity of 73.0% (Figure 2). Applying a random-effects model yielded a pooled reversal rate of 0.834 (95% CI: 0.709–0.933). The source of high heterogeneity was further examined by exploring type of HRS, age, and the region of the study (Western versus Asian). Only four [7, 19, 24, 31] and two [23, 29] out of eight studies reported the reversal rate of type I and type II HRS, respectively. Although the reversal rate was still highly varied (range: 0.333 to 0.938) in type I HRS, it was less varied in type II HRS (range: 0.667 to 0.881) with the *I*^2^ of 83.5% and 0%, respectively. The reversal rate was a bit lower in type I HRS than type II HRS with the pooled reversal rate of 0.702 (95% CI: 0.468–0.894) versus 0.860 (95% CI: 0.741–0.950), although this was not significant (Figures 3 and 4).

Neither age group nor region was detected as the source of heterogeneity. As for a subgroup analysis by age groups ≥50 versus <50 years, the degree of heterogeneity *I*^2^ was 76.6% and 74.3%, respectively, with not much different reversal rates of 0.811 (95% CI: 0.641–0.940) versus 0.859 (95% CI: 0.625–0.996) (Figure S1). Six studies [7, 15, 19, 23, 28, 29] were conducted in Western countries and two [21, 31] were conducted in Asian countries where reversal rates were still highly heterogeneous in both regions with the rates of 0.819 (95% CI: 0.660–0.937) and 0.920 (95% CI: 0.801–0.993), respectively (Figure S2). Among eight studies that reported HRS reversal and the pretransplant treatment with vasoconstrictor plus albumin, requirement and duration of hemodialysis could not be evaluated due to inadequate details of data. In addition, we investigated publication bias, but neither Egger's test (coefficient = 0.61, standard error = 0.92, and* P* = 0.533) nor the funnel plot (Figure S3) suggested bias from missing studies.

### 3.4. Death Rate

A total of 17 of the 20 studies reported death rates [7, 13, 14, 16–18, 20–27, 29–31], and the data were extracted and showed as number of at risk patients (Table S2). Among these studies, 11 compared death rates in HRS patients and non-HRS patients and had sample sizes of 8–130 and 15–1163, respectively. These studies had follow-up periods of 1–10 years. The aggregated data of these 17 studies were then expanded to individual patient data.

**Figure 5:**
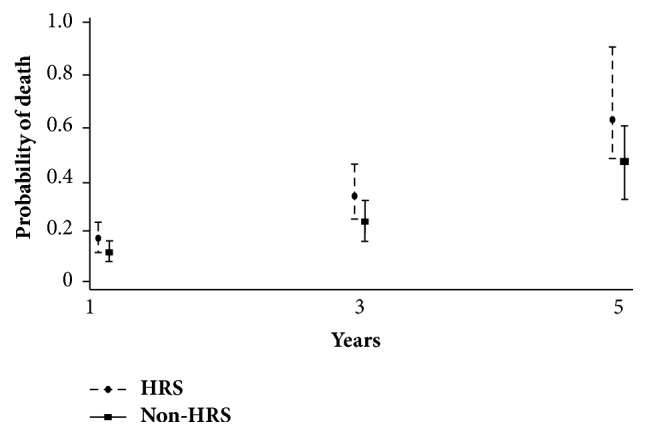
Probability of death at 1, 3, and 5 years in hepatorenal syndrome (HRS) versus non-HRS groups.

Poisson regression analysis was applied by fitting time and HRS versus non-HRS in the equation. The probability of death at 1, 3, and 5 years was estimated and plotted. The probability of death tended to be higher in HRS patients than in non-HRS patients (Figure 5). The pooled overall death rates were 0.25 (95% CI: 0.18–0.33) and 0.19 (95% CI: 0.14–0.26) for HRS patients and non-HRS patients after LT, respectively, yielding a risk ratio of death of 1.29 (95% CI: 1.14–1.47, *P* < 0.001). This finding indicated that HRS patients were 1.29 times more likely to die after LT than non-HRS patients.

Due to inadequate data of the 20 recruited studies, the effects of clinical and laboratory parameters, e.g., the duration of HRS, hemodialysis requirement, vasopressor use, baseline serum creatinine level, waiting time prior to LT, and types of immunosuppressive regimens on the outcomes of HRS could not be explored. The type of HRS was the only factor with which we next performed subgroup analysis (Table 2). We found that the one-year posttransplant death rate for type I HRS was slightly higher than type II HRS (0.250; 95% CI: 0.166–0.377 versus 0.159; 95% CI: 0.100–0.253). However, the death rates were not much different at 3 and 5 years: 0.344 (95% CI: 0.217–0.547) versus 0.316 (95% CI: 0.201–0.497) and 0.494 (95% CI: 0.307–0.793) versus 0.406 (95% CI: 0.249–0.661), respectively.

### 3.5. Incidence of Acute Cellular Rejection

**Figure 6:**
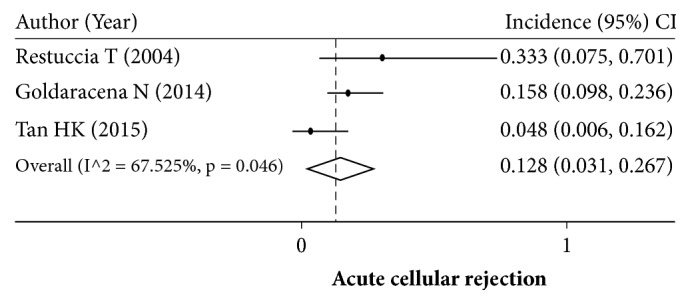
Pooling incidence of acute cellular rejection.

Only 3 studies reported ACR, and the rate of ACR ranged from 0.048 to 0.333. The overall incidence of ACR was 0.128 (95% CI: 0.031–0.267) (Figure 6).

## 4. Discussion

Our meta-analysis demonstrated that about 83% of HRS patients achieved HRS reversal after LT. A recent meta-analysis study reported a precise pooled estimation of only 49.5% for HRS reversal after a combination of vasoconstrictors with albumin infusion treatment [32]. The use of pharmacotherapy in HRS is beneficial in less than 50% of patients and it is not a highly effective treatment for HRS. Moreover, there was no reduction in mortality from medical therapy [32]. Therefore, LT should be performed in decompensated cirrhotic patients with HRS, particularly when pharmacotherapy fails. Although our meta-analysis revealed that the death rate of LT patients with HRS was higher than that of LT patients without HRS (i.e., 25% versus 19%), high MELD score and other causes can be concomitant risk factors of posttransplant death. Patients with HRS who failed pharmacotherapy should undergo LT in a short period of time.

Our pooling was characterized by high heterogeneity. Accordingly, we attempted to assess the source of heterogeneity by performing subgroup analysis based on the type of HRS, the location of the study, and mean age of patients (≥50 versus <50 years). However, none of these factors could be identified as the source of the heterogeneity. Subgroup analysis demonstrated lower rates of HRS reversal in type I HRS patients, older age group, and Western population with high heterogeneity. We were also interested in MELD score, pretransplant vasoconstrictor use, requirement, and duration of hemodialysis, but there were inadequate data to perform subgroup analysis among these parameters.

Interestingly, our finding of an estimated 83% HRS reversal rate indicated that 17% of patients who underwent LT did not achieve HRS reversal after LT. Sharma et al. reported that a lower GFR after LT was one of the predictors for post-LT mortality [33]. As a result, patients with HRS nonreversal after LT could have an increased mortality. HRS reversal in the early period post-LT reflects better survival and good outcomes. Our study demonstrated that the 1-year, 3-year, 5-year, and overall death rates after LT were higher for HRS patients than non-HRS patients. In addition to having lower chance of HRS reversal, patients with type I HRS had higher risk of death than type II HRS patients resulting in a poorer prognosis of type I HRS after LT. Although HRS reversal occurred in the majority of patients, there were some risks that renal function might be persistently impaired. Due to the use of calcineurin inhibitors, the kidneys of HRS patients may subject to further impaired renal function. The rate of chronic kidney disease was reported at 6–16% up to 5 years after LT in HRS patients [17, 20].

In the present study, approximately 12.8% of HRS patients experienced ACR. In 2004, Restuccia et al. reported a rather high incidence of ACR in HRS patients [23], and two later studies reported lower incidence of ACR [17, 29]. The reason for these contradictory reports of ACR rates in LT with HRS is unknown, but the lower ACR rates in the latter studies might be associated with more effective immunosuppressive agents, an improvement in surgical techniques, and better postoperative care. The pooled incidence rate of ACR in HRS patients in the present study was not different from previous reports (9–46%) [34].

## 5. Conclusion

This meta-analysis is the first to demonstrate the posttransplant outcomes of HRS patients. Over 80% of HRS cases are reversible after LT. The mortality rates of patients following transplantation are higher than the patients who underwent LT without HRS but inadequate data, heterogeneous-type of populations, and being observational studies added some limitation to our study. However, most of the recruited studies were rated as having fair to good quality based on the Newcastle-Ottawa scale [11].

## Figures and Tables

**Table 1:** Characteristic of including studies.

Authors	Year	Country	IAC criteria	Type of study	Period of study	Donor type^*∗*^	N	Age^*∗∗*^	MELD^#^	Cr^†^
Briceño et al. [13]	2011	Spain	1996 & 2007	Retrospective	1995–2008	DDLT	66	51	25	2.1
Boyer et al. [14]	2011	USA, Germany	1996	RCT	NA	DDLT	35	NA	32	NA
Cassinello et al. [15]	2003	Spain	1996	Prospective	NA	DDLT	10	46	NA	2.2
Chok et al. [16]	2012	Hong Kong	1996 & 2007	Prospective	1997–2007	LDLT	33	52	43	2.8
Goldaracena et al. [17]	2014	Canada	2007	Prospective	2000–2012	DDLT, LDLT	120	52	32	2.9
Lee et al. [18]	2012	Korea	1996	Retrospective	2000–2010	DDLT, LDLT	71	50	38	3.0
Marik et al. [19]	2006	USA	1996	Prospective	2001–2004	DDLT, LDLT	28	51	30	2.9
Nadim et al. [20]	2012	USA	1996	Retrospective	2002–2006	DDLT	35	50	40	NA
Park et al. [21]	2010	Korea	2007	Prospective	2005–2008	DDLT	8	46	33	3.2
Park et al. [22]	2015	Korea	1996	Retrospective	2005–2012	DDLT, LDLT	76	52	38	3.0
Restuccia et al. [23]	2004	Spain	1996	Prospective	1996–2010	DDLT	9	50	NA	2.7
Rice et al. [24]	2011	USA	2007	Retrospective	1997–2004	DDLT	43	53	32	NA
Rodriguez et al. [25]	2015	Spain	1996	Retrospective	1998–2014	DDLT	31	58	21	1.8
Ruiz et al. [26]	2006	USA	1996	Retrospective	1988–2004	DDLT	80	49	26	NA
Ruiz et al. [27]	2007	USA	1996	Prospective	1995–2004	DDLT	130	49	34	NA
Shusterman et al. [28]	2007	USA	1996	Retrospective	1999–2005	DDLT	17	47	NA	NA
Tan et al. [29]	2015	Canada	2007	Retrospective	2000–2012	DDLT	42	58	21	1.8
Wong et al. [7]	2015	Canada	2007	Retrospective	2001–2010	DDLT	62	55	35	3.3
Xing et al. [30]	2013	China	1996	Prospective	2001–2009	DDLT	18	46	25	2.8
Xu et al. [31]	2009	China	1996	Prospective	2003–2006	DDLT	21	47	33	3

^*∗*^IAC: International Ascites Club; N: number; DDLT: deceased donor liver transplantation; LDLT: living donor liver transplantation; NA: non-applicable. ^*∗∗*^Age: expressed as mean. ^#^MELD: model of end stage liver disease, expressed as mean. ^†^Cr: creatinine, expressed as mean (mg/dL).

**Table 2:** Estimation of 1-, 3-, and 5-year post-liver transplant death rate based on the presence of hepatorenal syndrome (HRS) versus non-HRS and the type of HRS.

Patient	Death rate (person-year)					
	1-year	95% CI	3-year	95% CI	5-year	95% CI
*Presence of HRS*						
HRS	0.1707	0.1254–0.2324	0.3494	0.2553–0.4781	0.6487	0.4733–0.8890
Non-HRS	0.1186	0.0868–0.1621	0.2428	0.1770–0.3330	0.4508	0.3287–0.6181
*Type of HRS*						
Type 1 HRS	0.2502	0.1660–0.3773	0.3443	0.2166–0.5472	0.4936	0.3072–0.7929
Type 2 HRS	0.1592	0.1002–0.2530	0.3156	0.2005–0.4968	0.4055	0.2489–0.6607

## Supplementary Materials

Supplementary Materials Figure S1: pooling incidence of hepatorenal syndrome reversal as a function of age. Figure S2: pooling incidence of hepatorenal syndrome reversal as a function of geographic region. Figure S3: funnel plot of reports of hepatorenal syndrome reversal rates. Table S1: Newcastle-Ottawa quality assessment scale of each included study. Table S2: number of survivors in hepatorenal syndrome (HRS) groups versus non-HRS groups and the rate of HRS reversal and ACR in the HRS group.

## References

[1] Zipprich A., Garcia-Tsao G., Rogowski S., Fleig W. E., Seufferlein T., Dollinger M. M. (2012). Prognostic indicators of survival in patients with compensated and decompensated cirrhosis. *Liver International*.

[2] Arroyo V., Ginès P., Gerbes A. L. (1996). Definition and diagnostic criteria of refractory ascites and hepatorenal syndrome in cirrhosis. *Hepatology*.

[3] Salerno F., Gerbes A., Ginès P., Wong F., Arroyo V. (2007). Diagnosis, prevention and treatment of hepatorenal syndrome in cirrhosis. *Gut*.

[4] Angeli P., Gines P., Wong F. (2015). Diagnosis and management of acute kidney injury in patients with cirrhosis: Revised consensus recommendations of the International Club of Ascites. *Gut*.

[5] Ginès P. Q. E., Arroyo V. (2010). EASL clinical practice guidelines on the management of ascites. *Journal of Hepatology*.

[6] Fabrizi F., Aghemo A., Messa P. (2013). Hepatorenal syndrome and novel advances in its management. *Kidney and Blood Pressure Research*.

[7] Wong F., Leung W., Al Beshir M., Marquez M., Renner E. L. (2015). Outcomes of patients with cirrhosis and hepatorenal syndrome type 1 treated with liver transplantation. *Liver Transplantation*.

[8] Shamseer L., Moher D., Clarke M. (2015). Preferred reporting items for systematic review and meta-analysis protocols (prisma-p) 2015: Elaboration and explanation. *British Medical Journal*.

[9] Booth A., Clarke M., Dooley G. (2012). The nuts and bolts of PROSPERO: An international prospective register of systematic reviews. *Systematic Reviews*.

[10] Salerno F., Gerbes A., Gines P., Wong F., Arroyo V. (2008). Diagnosis, prevention and treatment of hepatorenal syndrome in cirrhosis. *Postgraduate Medical Journal*.

[11] Wells B. S., O'Connell D., Peterson J., Welch V., Losos M., Tugwell P. The Newcastle-Ottawa Scale (NOS) for assessing the quality of nonrandomised studies in meta-analyses.

[12] Thakkinstian A., Ingsathit A., Chaiprasert A. (2011). A simplified clinical prediction score of chronic kidney disease: A cross-sectional-survey study. *BMC Nephrology*.

[13] Briceño J., Ciria R., de la Mata M., Montero J. L., Rufián S., López-Cillero P. (2011). Extended criteria donors in liver transplant candidates with hepatorenal syndrome. *Clinical Transplantation*.

[14] Boyer T. D., Sanyal A. J., Garcia-Tsao G. (2011). Impact of liver transplantation on the survival of patients treated for hepatorenal syndrome type 1. *Liver Transplantation*.

[15] Cassinello C., Moreno E., Gozalo A., Ortuño B., Cuenca B., Solís-Herruzo J. A. (2003). Effects of Orthotopic liver transplantation on vasoactive systems and renal function in patients with advanced liver cirrhosis. *Digestive Diseases and Sciences*.

[16] Chok K. S. H., Fung J. Y. Y., Chan S. C. (2012). Outcomes of living donor liver transplantation for patients with preoperative type 1 hepatorenal syndrome and acute hepatic decompensation. *Liver Transplantation*.

[17] Goldaracena N., Marquez M., Selzner N. (2014). Living vs. deceased donor liver transplantation provides comparable recovery of renal function in patients with hepatorenal syndrome: A matched case-control study. *American Journal of Transplantation*.

[18] Lee J. P., Kwon H. Y., Park J. I. (2012). Clinical outcomes of patients with hepatorenal syndrome after living donor liver transplantation. *Liver Transplantation*.

[19] Marik P. E., Wood K., Starzl T. E. (2006). The course of type 1 hepato-renal syndrome post liver transplantation. *Nephrology Dialysis Transplantation *.

[20] Nadim M. K., Genyk Y. S., Tokin C. (2012). Impact of the etiology of acute kidney injury on outcomes following liver transplantation: Acute tubular necrosis versus hepatorenal syndrome. *Liver Transplantation*.

[21] Park I., Moon E., Hwang J.-A. (2010). Does hepatorenal syndrome affect the result of liver transplantation? Clinical observations. *Transplantation Proceedings*.

[22] Park J. Y., Gwak G. Y., Kim J. M. (2015). Renal outcomes after liver transplantation in fulminant hepatitis a with acute kidney injury: Comparison with hepatorenal syndrome. *Transplantation Proceedings*.

[23] Restuccia T., Ortega R., Guevara M. (2004). Effects of treatment of hepatorenal syndrome before transplantation on posttransplantation outcome. A case-control study. *Journal of Hepatology*.

[24] Rice J. P., Skagen C., Said A. (2011). Liver transplant outcomes for patients with hepatorenal syndrome treated with pretransplant vasoconstrictors and albumin. *Transplantation*.

[25] Rodriguez E., Henrique Pereira G., Solà E. (2015). Treatment of type 2 hepatorenal syndrome in patients awaiting transplantation: Effects on kidney function and transplantation outcomes. *Liver Transplantation*.

[26] Ruiz R., Kunitake H., Wilkinson A. H. (2006). Long-term analysis of combined liver and kidney transplantation at a single center. *JAMA Surgery*.

[27] Ruiz R., Barri Y. M., Jennings L. W. (2007). Hepatorenal syndrome: A proposal for kidney after liver transplantation (KALT). *Liver Transplantation*.

[28] Shusterman B., Mchedishvili G., Rosner M. H. (2007). Outcomes for Hepatorenal Syndrome and Acute Kidney Injury in Patients Undergoing Liver Transplantation: A Single-Center Experience. *Transplantation Proceedings*.

[29] Tan H. K., Marquez M., Wong F., Renner E. L. (2015). Pretransplant Type 2 Hepatorenal Syndrome Is Associated with Persistently Impaired Renal Function after Liver Transplantation. *Transplantation*.

[30] Xing T., Zhong L., Chen D., Peng Z. (2013). Erratum: Experience of combined liver-kidney transplantation for acute-on-chronic liver failure patients with renal dysfunction (Transplantation Proceedings (2013) 45:6 (2307-2313)). *Transplantation Proceedings*.

[31] Xu X., Ling Q., Zhang M. (2009). Outcome of patients with hepatorenal syndrome type 1 after liver transplantation: Hangzhou experience. *Transplantation*.

[32] Salerno F., Navickis R. J., Wilkes M. M. (2015). Albumin treatment regimen for type 1 hepatorenal syndrome: A dose-response meta-analysis. *BMC Gastroenterology*.

[33] Sharma P., Welch K., Eikstadt R., Marrero J. A., Fontana R. J., Lok A. S. (2009). Renal outcomes after liver transplantation in the model for end-stage liver disease era. *Liver Transplantation*.

[34] Shaked A., Ghobrial R. M., Merion R. M. (2009). Incidence and severity of acute cellular rejection in recipients undergoing adult living donor or deceased donor liver transplantation. *American Journal of Transplantation*.

[35] Utako P., Emyoo T., Anothaisintawee T., Yamashiki N., Thakkinstian A., Sobhonslidsuk A. (2017). Clinical Outcomes after Liver Transplantation for Hepatorenal Syndrome: A Systematic Review and Metaanalysis. *Gastroenterology*.

[36] Utako P., Emyoo T., Anothaisintawee T., Yamashiki N., Thakkinstian A., Sobhonslidsuk A. (2017). Clinical Outcomes after Liver Transplantation for Hepatorenal Syndrome: A Systematic Review and Metaanalysis. *Gastroenterology*.

