# Supplementary material for: Clinical Outcomes after Liver Transplantation for Hepatorenal Syndrome: A Systematic Review and Meta-Analysis
Source: Biomed Res Int. 2018 May 24;2018:5362810. doi: 10.1155/2018/5362810 (PMC5994306; doi:10.1155/2018/5362810)
Supplement: Supplementary Materials — Figure S1: pooling incidence of hepatorenal syndrome reversal as a function of age. Figure S2: pooling incidence of hepatorenal syndrome reversal as a function of geographic region. Figure S3: funnel plot of reports of hepatorenal syndrome reversal rates. Table S1: Newcastle-Ottawa quality assessment scale of each included study. Table S2: number of survivors in hepatorenal syndrome (HRS) groups versus non-HRS groups and the rate of HRS reversal and ACR in the HRS group. [file 5362810.f1.docx]

**
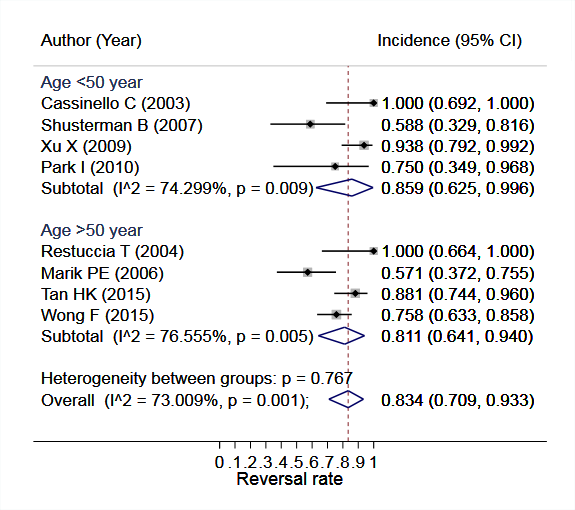
**

**S1 Figure:** Pooling incidence of hepatorenal syndrome reversal as a function of age


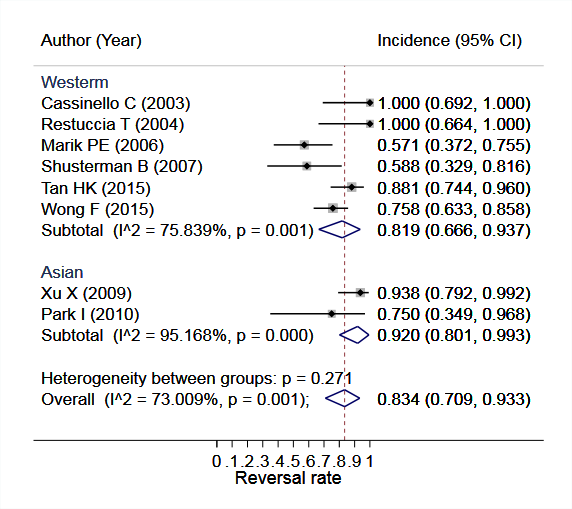


**S2 Figure:** Pooling incidence of hepatorenal syndrome reversal as a function of geographic region


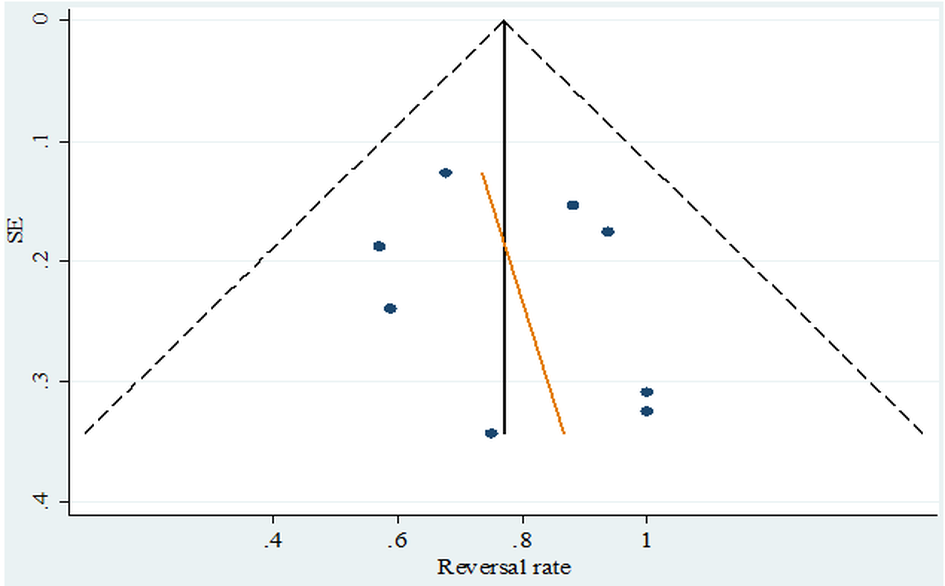


**S3 Figure:** Funnel plot of reports of hepatorenal syndrome reversal rates

**S1 Table:** Newcastle-Ottawa quality assessment scale of each included studies.

| **Author** | **Year** | S1 | S2 | S3 | S4 | C | O1 | O2 | O3 |
| --- | --- | --- | --- | --- | --- | --- | --- | --- | --- |
| Briceno J et al.[[28](#_ENREF_28)] | 2011 | **🟑** | **🟑** | **🟑** | **🟑** | **🟑** | **🟑** | **🟑** |  |
| Cassinello C et al.[[35](#_ENREF_35)] | 2003 |  | **🟑** | **🟑** | **🟑** |  | **🟑** | **🟑** | **🟑** |
| Chok KS et al.[[26](#_ENREF_26)] | 2012 | **🟑** | **🟑** | **🟑** | **🟑** |  | **🟑** | **🟑** | **🟑** |
| Goldaracena N et al.[[16](#_ENREF_16)] | 2014 | **🟑** | **🟑** | **🟑** | **🟑** | **🟑🟑** | **🟑** | **🟑** | **🟑** |
| Lee JP et al.[[25](#_ENREF_25)] | 2012 | **🟑** | **🟑** | **🟑** | **🟑** |  |  | **🟑** | **🟑** |
| Marik PE et al.[[33](#_ENREF_33)] | 2006 | **🟑** | **🟑** | **🟑** | **🟑** | **🟑** | **🟑** | **🟑** |  |
| Nadim MK et al.[[24](#_ENREF_24)] | 2012 | **🟑** | **🟑** | **🟑** | **🟑** |  |  | **🟑** | **🟑** |
| Park I et al.[[7](#_ENREF_7)] | 2010 | **🟑** | **🟑** | **🟑** | **🟑** |  | **🟑** | **🟑** | **🟑** |
| Park JY et al.[[22](#_ENREF_22)] | 2015 | **🟑** | **🟑** | **🟑** | **🟑** |  |  | **🟑** | **🟑** |
| Restuccia T et al.[[34](#_ENREF_34)] | 2004 | **🟑** | **🟑** | **🟑** | **🟑** | **🟑🟑** | **🟑** | **🟑** | **🟑** |
| Rice JP et al.[[27](#_ENREF_27)] | 2011 | **🟑** | **🟑** | **🟑** | **🟑** |  | **🟑** | **🟑** | **🟑** |
| Rodriguez E et al.[[21](#_ENREF_21)] | 2015 | **🟑** | **🟑** | **🟑** | **🟑** |  | **🟑** | **🟑** | **🟑** |
| Ruiz R et al.[[31](#_ENREF_31)] | 2007 | **🟑** | **🟑** | **🟑** | **🟑** |  |  | **🟑** | **🟑** |
| Shusterman B et al.[[30](#_ENREF_30)] | 2007 | **🟑** | **🟑** | **🟑** | **🟑** | **🟑** | **🟑** | **🟑** | **🟑** |
| Tan HK et al.[[20](#_ENREF_20)] | 2015 | **🟑** | **🟑** | **🟑** | **🟑** | **🟑🟑** | **🟑** | **🟑** | **🟑** |
| Wong F et al.[[12](#_ENREF_12)] | 2015 | **🟑** | **🟑** | **🟑** | **🟑** |  |  | **🟑** | **🟑** |
| Xing T et al.[[23](#_ENREF_23)] | 2013 | **🟑** | **🟑** | **🟑** | **🟑** |  | **🟑** | **🟑** | **🟑** |
| Xu X et al.[[29](#_ENREF_29)] | 2009 | **🟑** | **🟑** | **🟑** | **🟑** |  | **🟑** | **🟑** | **🟑** |
| Ruiz R et al.[[32](#_ENREF_32)] | 2006 | **🟑** | **🟑** | **🟑** | **🟑** | **🟑** | **🟑** | **🟑** | **🟑** |

S, selection; C, comparability; O, outcome

**S2 Table**: Number of survivors in hepatorenal syndrome (HRS) groups versus non-HRS groups, and the rate of HRS reversal and ACR in the HRS group

| Authors | Year | N survivor HRS^*^ | | | | N survivor non-HRS^*^ | | | |  |  |
| --- | --- | --- | --- | --- | --- | --- | --- | --- | --- | --- | --- |
|  |  | N total | 1 | 3 | 5 | N total | 1 | 3 | 5 | N Reversal^#^ | N ACR^†^ |
| Boyer T et al. | 2011 | 35 | 20 |  |  |  |  |  |  |  |  |
| Briceno J et al. | 2011 | 66 | 54 | 31 | 13 | 432 | 359 | 241 | 136 |  |  |
| Cassinello C et al. | 2003 | 10 |  |  |  |  |  |  |  | 10 |  |
| Chok KS et al. | 2012 | 33 | 29 | 22 | 15 | 71 | 70 | 61 | 38 |  |  |
| Goldaracena N et al. | 2014 | 120 | 98 | 59 | 34 |  |  |  |  |  | 19 |
| Lee JP et al. | 2012 | 71 | 57 | 35 |  | 655 | 594 | 460 | 86 |  |  |
| Marik PE et al. | 2006 | 28 |  |  |  |  |  |  |  | 16 |  |
| Nadim MK et al. | 2012 | 35 | 31 | 21 | 12 | 248 | 207 | 141 |  |  |  |
| Park I et al. | 2010 | 8 | 7 |  |  | 63 | 60 |  |  | 6 |  |
| Park JY et al. | 2015 | 76 | 61 | 56 |  | 20 | 14 | 14 |  |  |  |
| Restuccia T et al. | 2004 | 9 | 9 | 9 |  | 27 | 27 | 23 |  | 9 | 3 |
| Rice JP et al. | 2011 | 43 | 37 |  |  |  |  |  |  |  |  |
| Rodriguez E et al. | 2015 | 31 | 27 | 26 |  | 15 | 14 | 14 |  |  |  |
| Ruiz R et al | 2007 | 130 | 97 | 50 | 21 | 1163 | 1047 | 782 | 493 |  |  |
| Ruiz R et al. | 2006 | 80 | 53 |  |  |  |  |  |  |  |  |
| Shusterman B et al. | 2007 | 17 |  |  |  |  |  |  |  | 10 |  |
| Tan HK et al. | 2015 | 42 | 39 |  |  | 83 | 76 |  |  | 37 | 2 |
| Wong F et al. | 2015 | 62 | 54 | 50 | 50 |  |  |  |  | 47 |  |
| Xing T et al. | 2003 | 18 | 11 | 4 | 1 | 103 | 78 | 44 | 23 |  |  |
| Xu X et al. | 2009 | 32 | 21 |  |  |  |  |  |  | 30 |  |

*N survivor: number of survival population, expressed as person at 1, 3, 5 year

#N Reversal: number of population achieving HRS reversal

†N ACR: number of population having an episode of acute cellular rejection
